# A case of hypoglossal nerve palsy with evolving cranial nerve involvement in renal cell carcinoma: a case report

**DOI:** 10.1186/s13256-025-05193-3

**Published:** 2025-04-01

**Authors:** Shadi Shams, Abigail Broughton, Katrina Lambeth, Aditi Trivedi, Dehua Wang, Sun Choo, Katherine Dove

**Affiliations:** 1 Department of Pediatrics, University of California, San Diego, CA 92093 USA; 2 Department of Pediatrics, Rady Children’S Hospital, San Diego, CA 92123 USA; 3 Department of Neurosciences, Division of Child Neurology, University of California, San Diego, CA 92093 USA; 4 Department of Pediatrics, Division of Pediatric Hematology/Oncology, University of California, San Diego, CA 92093 USA; 5 Department of Pathology, Division of Pediatric Pathology and Hematopathology, University of California, San Diego, CA 92093 USA; 6 School of Medicine, University of California, San Diego, CA 92093 USA

**Keywords:** Skull base metastasis, Renal cell carcinoma, RCC, Hypoglossal nerve, Glossopharyngeal nerve, Multiple cranial neuropathies, Child neurology

## Abstract

**Background:**

Renal cell carcinoma is a rare pediatric solid tumor that typically presents with hematuria, abdominal mass, or flank pain. It is uncommon for renal cell carcinoma to manifest with headache and isolated extra-urogenital symptoms. We present, to our knowledge, the first case of renal cell carcinoma with bony metastases, presenting initially as isolated cranial nerve twelve palsy. Although bony metastases can occur in renal cell carcinoma, skull-based metastases and cranial neuropathies are exceedingly rare, especially in the pediatric population.

**Case presentation:**

We describe the unusual presentation of renal cell carcinoma with bony skull-based metastases presenting initially as isolated hypoglossal nerve palsy, that progressed to multiple cranial neuropathies in a previously healthy 14-year-old female of Indian descent.

**Conclusion:**

The differential for hypoglossal nerve with evolving cranial nerves 9 and 10 involvement can be broad owing to the course of the nerve, the structures surrounding it, and its pathway. It is important for providers to include bony metastatic disease in the differential diagnosis for headaches with multiple cranial neuropathies.

## Introduction

Hypoglossal nerve injuries are often associated with multiple cranial neuropathies, owing to their proximity to other cranial nerves [1]. An isolated hypoglossal nerve palsy is rarely reported and uncommon as an initial presenting sign. The differential for hypoglossal nerve palsy with evolving cranial nerve neuropathies remains broad and includes, among others, traumatic, infectious, vascular, and neoplastic etiologies. Renal cell carcinoma (RCC) is a prevalent urogenital cancer in adults. However, it rarely occurs in the pediatric population, with an incidence of approximately 0.1–0.3% among all pediatric neoplasms [2, 3]. The most common presenting symptoms include hematuria, abdominal mass, or flank pain. However, the symptoms can be heterogeneous, and some patients may remain asymptomatic initially [4]. According to data reported in adults, RCC tends to metastasize to the liver, lung, brain, adrenal gland, and bone [5]. RCC metastasis to the brain is relatively rare and occurs in 4–11% of cases in the span of 1–5 years from the onset of renal symptoms [6]. Once metastasis has occurred, patients may develop atypical site-specific symptoms in addition to the common presenting symptoms [6]. Clivus and skull-based metastases are exceedingly rare in the pediatric population [7], and clival metastasis with hypoglossal nerve involvement has been reported only once in the adult population [8].

Here we report the case of a 14-year-old female of Indian descent who presented with unilateral tongue deviation, dysphagia, uvular deviation, and headache. She was found to have an aggressive infiltrative osseous lesion on the clivus of the left occipital bone near the hypoglossal canal. The patient was ultimately diagnosed with transcription factor E3 (TFE3)-rearranged RCC and started on an appropriate chemotherapy regimen. TFE3 RCC is a rare subtype of RCC, but the most common type seen in children. To date, this unique presentation of RCC has yet to be reported in literature. Through this case report, we hope to shed light on the importance of considering bony metastases to the skull base in the differential of multiple cranial neuropathies, especially since this would lead to important potential treatment options and prognostication discussions.

## Case presentation

A 14-year-old previously healthy female of Indian descent presented with 3 weeks of persistent, mild left posterior auricular achy headache, gradually progressive left-sided neck pain, and dysphagia without any dysarthria. A review of systems was negative for fever, weight loss, vision or hearing changes, weakness, changes in sensation, neck stiffness, abdominal pain, and hematuria. The initial exam showed an isolated left-sided tongue deviation. Given the nummular character of her headache, as well as dysphagia and uvular deviation, there was suspicion of hypoglossal nerve impairment, and suggestion of multiple cranial neuropathies, initiating a comprehensive workup. Dysphagia study showed that dysphagia is secondary to oropharyngeal impairment.

**Fig. 1:**
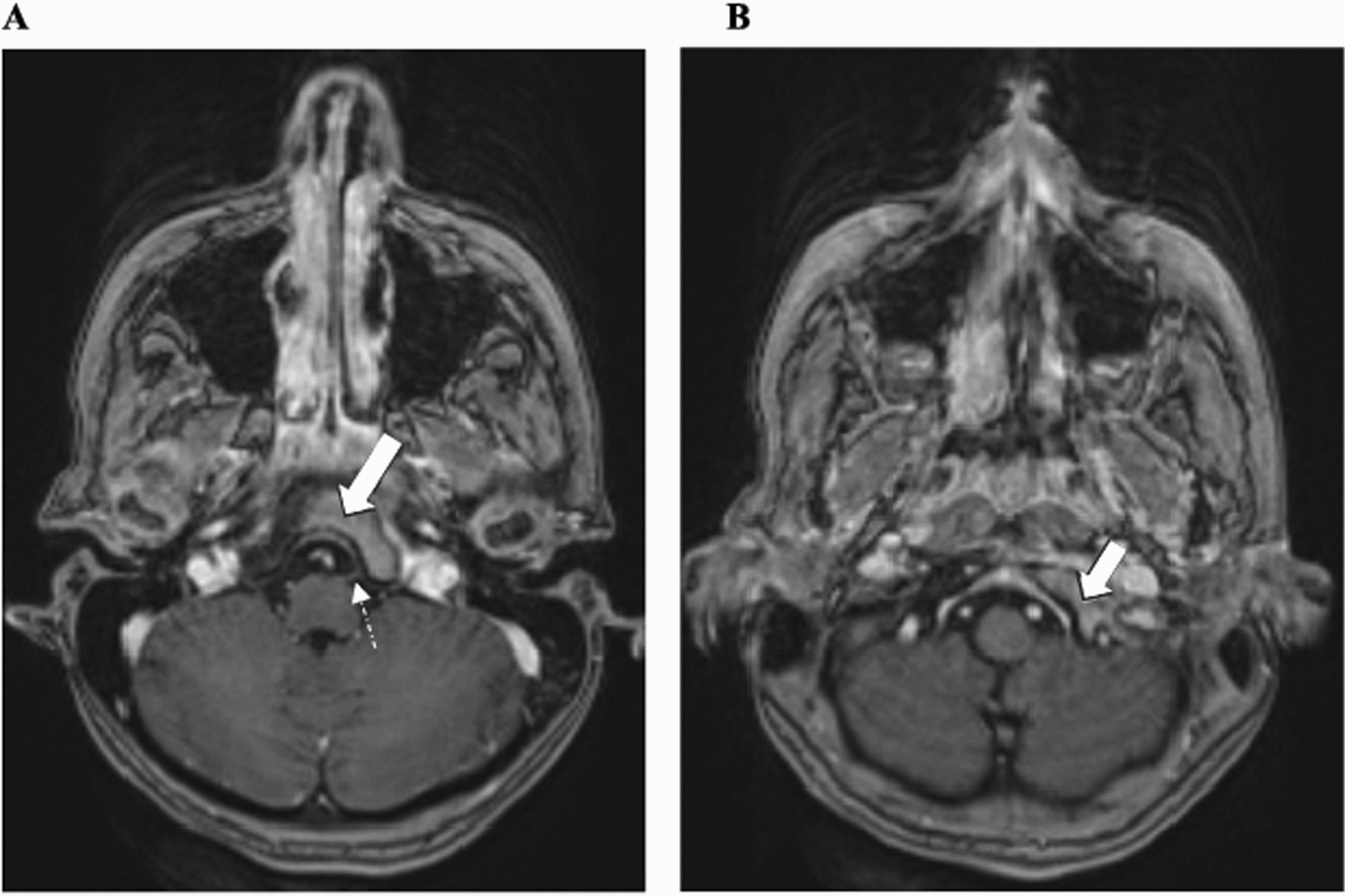
Magnetic resonance imaging with fast imaging employing a steady-state acquisition sequencing indicating the involvement of an aggressive infiltrative osseous lesion on the clivus of the left occipital bone near the hypoglossal canal. (**A**) Asymmetric left clivus bone enhancement. The left hypoglossal nerve can be visualized and is non-enhancing (exiting the ventral medulla, marked by a dotted arrow), (**B**) demonstrating asymmetric left occipital bone enhancement compared with the right

**Fig. 2:**
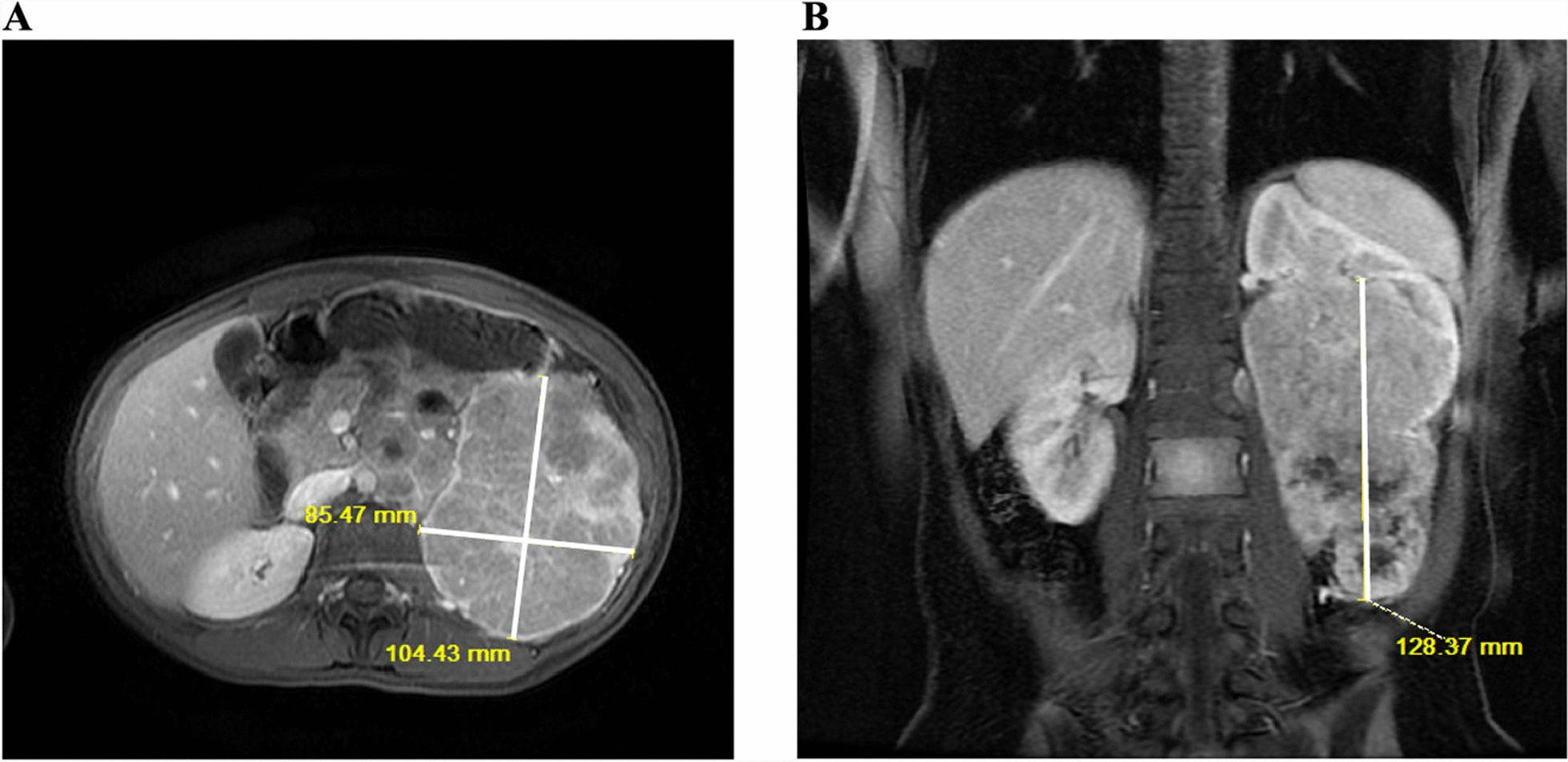
Magnetic resonance imaging of the abdomen and pelvis shows a large left-sided renal mass. (**A**) Axial and (**B**) coronal sequences are displayed

Laboratory tests were significant only for an elevated erythrocyte sedimentation rate of 43 mm/hour and mild normocytic anemia with a hemoglobin of 11.2 g/dL. The complete blood count, comprehensive metabolic panel, C-reactive protein, and urinalysis were otherwise within normal limits. For further evaluation of chronic, progressive headaches with bulbar symptoms, several imaging studies were performed. She had brain magnetic resonance imaging (MRI), a MRI cervical spine, and a computed tomography (CT) angiogram of the head and neck, all with and without contrast. These studies were all normal aside from an incidentally noted hypoattenuating left thyroid nodule. Cerebrospinal fluid (CSF) studies yielded a normal opening pressure, cell count, glucose, and protein, with no bacterial growth on CSF culture. During the second day of her hospital stay, the patient demonstrated vague left-sided abdominal pain that she reported had been intermittently present for several months. She also reported nausea that resolved with food consumption and mild intermittent back pain. Her abdominal exam at that time was unremarkable. Her exam evolved to demonstrate left-sided uvular deviation in addition to tongue deviation. Initial symptoms, such as tongue deviation, posterior auricular headache, and dysphagia, raised concern for multiple cranial nerve involvements. Given the evolving exam with multiple cranial neuropathies, the suspicion of a skull base lesion was high, thus, an MRI brain (with and without contrast) with fast imaging employing a steady-state acquisition (FIESTA) sequence was performed 2 days following the first imaging for close evaluation of the skull base. As suspected, the images revealed an aggressive infiltrative osseous lesion on the clivus of the left occipital bone near the hypoglossal canal (Fig. 1). MRI of the entire spine was performed to evaluate infectious or metastatic disease throughout the remainder of her central nervous system. Spinal imaging showed multifocal osseous metastatic deposits on the T3, T8, and L3 vertebral bodies, in addition to a large left renal mass measuring 10 cm and extending towards the midline, which was suspicious for a primary renal neoplasm (Fig. 2).

**Fig. 3:**
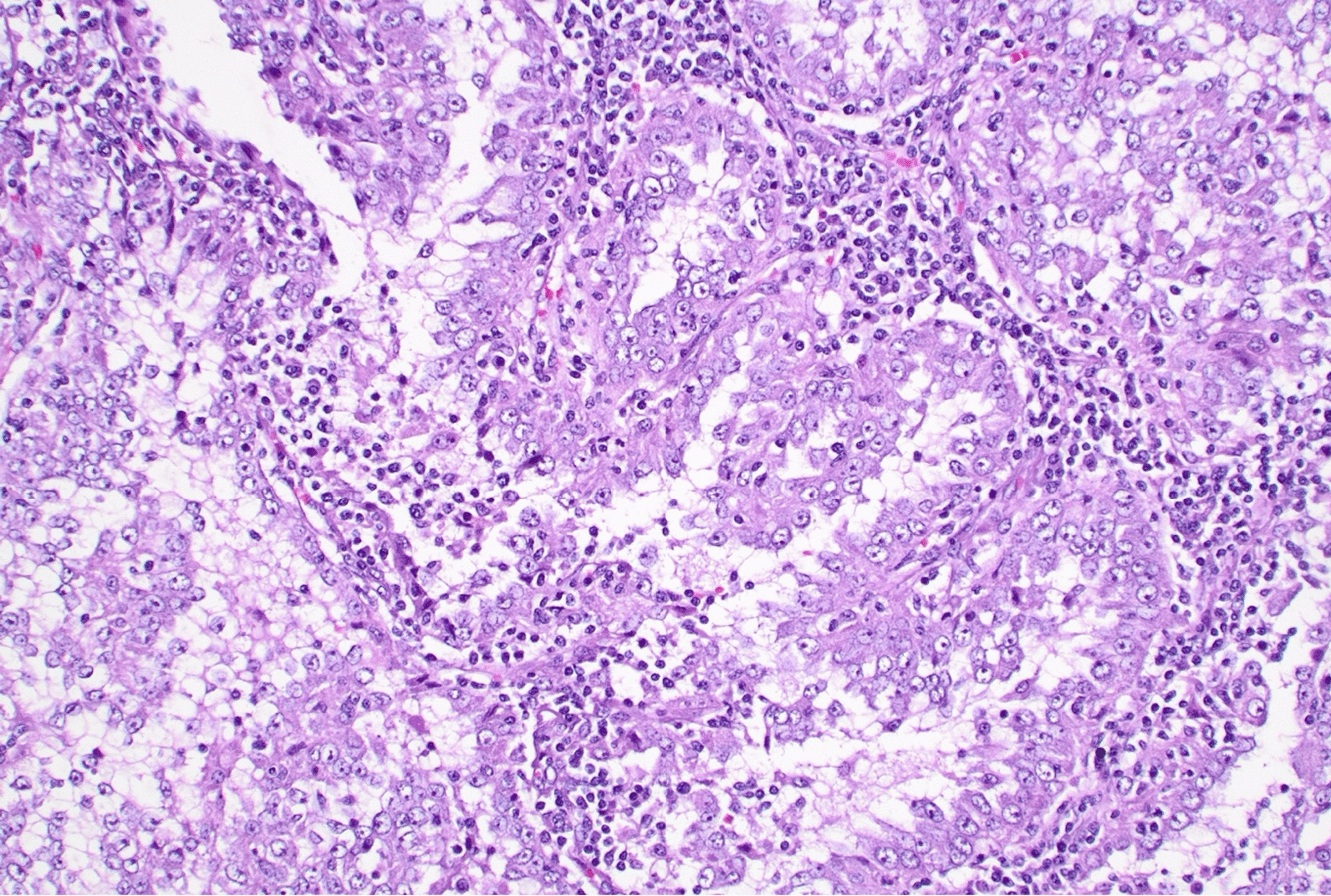
Histopathology of renal cell carcinoma biopsy site of primary renal tumor. The polygonal cells with clear cytoplasm and prominent nucleoli are the cancer cells that encase the majority of cells in the field

MRI of the abdomen and pelvis showed a 10.4 cm × 8.5 cm × 12.8 cm malignant left renal mass with metastatic retroperitoneal lymphadenopathy and bony metastasis to the right posterior acetabulum, ischial tuberosity, and the L3 vertebral body. A large retroperitoneal metastatic lymph node conglomerate was noted, causing severe compression of the left renal vein without definitive evidence of tumor invasion into the vein. CT chest showed numerous lung nodules representing metastases. Urinalysis continued to be normal without evidence of hematuria. Given the multiple metastatic lesions and large renal tumor, she underwent a biopsy which confirmed TFE3-rearranged RCC (Xp11 translocation RCC) (Fig. 3).

The patient was initiated on 4 mg of dexamethasone every 8 hours while awaiting the final pathology report, which led to the transient resolution of the hypoglossal palsy. Once diagnosis was confirmed as TFE3 RCC she was quickly weaned off dexamethasone and started on disease-directed therapy. She was started on pembrolizumab and cabozantinib. She subsequently received proton therapy to the clivus, spine, and right ischium. She was not noted to have any additional cranial neuropathies at her most recent follow-up visit 4 months after diagnosis. Scans 4 months after starting therapy showed a complete treatment response of her pulmonary metastasis and a significant disease response of her primary kidney mass and other metastatic sites.

## Discussion

To our knowledge, this case is the first description of systemic metastatic RCC initially presenting as an isolated cranial neuropathy with an evolving exam. Given the ultra-rare clinical presentation, maintaining a broad differential in the evaluation of headaches and cranial nerve palsies is essential to prevent diagnosis delays, particularly when time-sensitive therapeutics and prognostication are involved.

RCC has a predilection for bone metastasis. However, metastasis to the clivus is exceptionally rare, especially in pediatric patients [9]. The patient’s development of unilateral nummular appearing posterior auricular headache and oropharyngeal neuromuscular dysphagia were consistent with glossopharyngeal, vagal, and hypoglossal nerve palsies. Notably, she lacked the typical constellation of symptoms common in RCC, such as hematuria, fatigue, and weight loss. Her case underscores the importance of considering skull base metastasis in the differential diagnosis of multiple cranial neuropathies, as well as the role of thorough imaging in promptly identifying underlying lesions.

Given the spectrum of hypoglossal nerve palsy etiologies, including tumors, carotid dissection, infection, stroke, multiple sclerosis, and Guillain-Barré neuropathy, as shown in Table 1, a thorough workup is crucial [10–14]. RCC presentation can be quite heterogeneous, with patients often remaining asymptomatic until late in the disease course [4]. Despite widespread metastases, this patient’s normal urinalysis further complicated the early recognition of RCC. Depending on the location of metastasis, patients may present with a range of clinical symptoms, from neurological deficits to bone pain and headaches necessitating tailored treatment based on symptoms, primary diagnosis, and metastasis site [15].

**Table 1:**
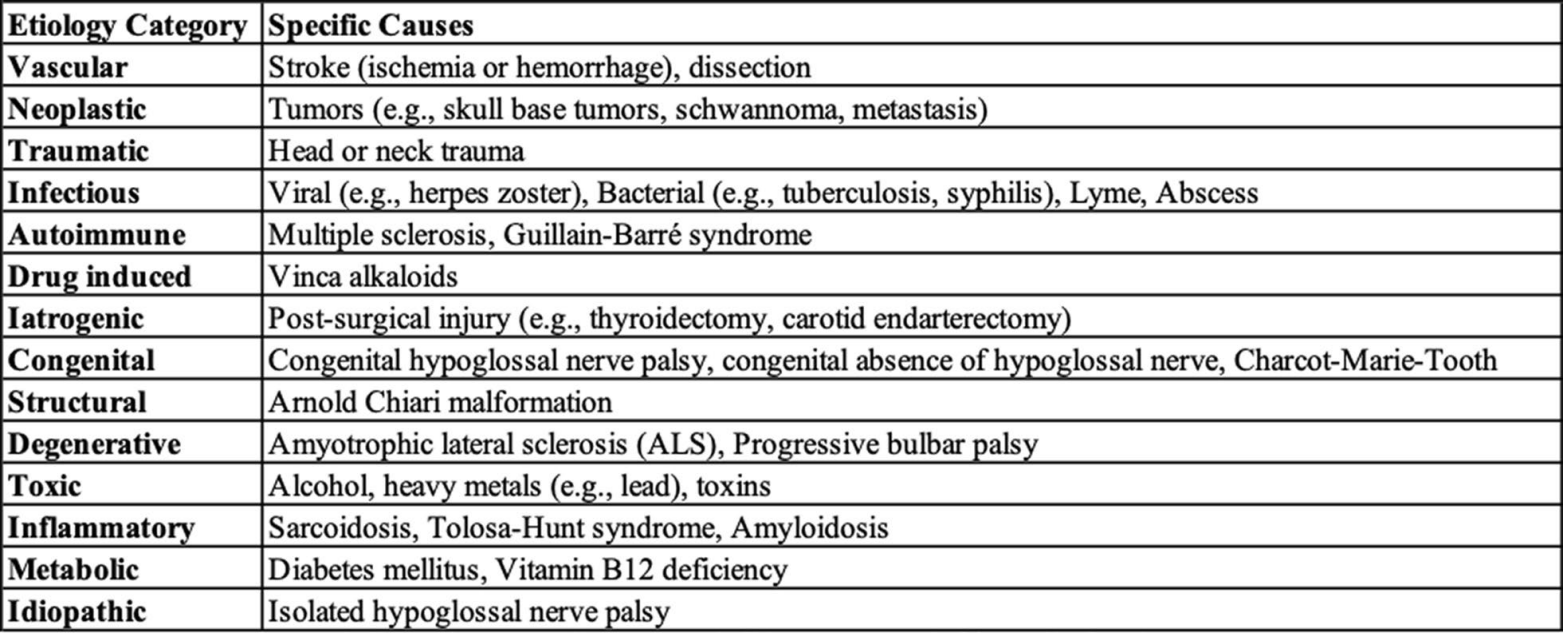
Summary of possible etiologies for hypoglossal nerve palsy

In addition, symptomatic steroid treatment for headaches may have masked the underlying malignancy, delaying diagnosis. Although treatment can vary depending on the RCC subtype and age of the patient, earlier diagnosis results in a shorter treatment period, better prognosis, improvement in patient’s quality of life, and increased chance of survival [16]. Given these challenges, we urge clinicians to recognize skull-base metastasis as a potential cause of multiple cranial nerve palsies. A systemic evaluation ruling out metastatic, infectious, autoimmune, traumatic, and demyelinating etiologies is imperative before considering a diagnosis of idiopathic nerve palsy.

## Conclusion

This case highlights the importance of maintaining a broad differential diagnosis for multiple cranial nerve palsies, which can result from neoplastic, vascular, infectious, inflammatory, or structural causes. The unique presentation of headache and cranial neuropathies due to skull base metastasis from an otherwise asymptomatic renal cell carcinoma underscores the critical role of thorough neurological examination and high-resolution diagnostic imaging of the cranial base and foramina. In addition, histopathological confirmation remains essential for accurate diagnosis and timely oncologic intervention, which can significantly improve patient outcomes.

## Data Availability

All data underlying the results are available as part of the article and no additional source data are required.

## Abbreviations

CSF: Cerebrospinal fluid
CT: Computed tomography
FIESTA: Fast imaging employing a steady-state acquisition
MRI: Magnetic resonance imaging
RCC: Renal cell carcinoma
TFE3: Transcription factor E3
